# The Signaling Pathways and Targets of Natural Compounds from Traditional Chinese Medicine in Treating Ischemic Stroke

**DOI:** 10.3390/molecules27103099

**Published:** 2022-05-12

**Authors:** Xing-Hua Li, Feng-Ting Yin, Xiao-Hang Zhou, Ai-Hua Zhang, Hui Sun, Guang-Li Yan, Xi-Jun Wang

**Affiliations:** 1 National Chinmedomics Research Center, National TCM Key Laboratory of Serum Pharmacochemistry, Heilongjiang University of Chinese Medicine, Harbin 150040, China; xinghualiabc@163.com (X.-H.L.); yinft177@163.com (F.-T.Y.); xiaohang20210101@163.com (X.-H.Z.); aihuatcm@163.com (A.-H.Z.); sunhui7045@163.com (H.S.); gancaosuan@163.com (G.-L.Y.); 2 State Key Laboratory of Quality Research in Chinese Medicine, Macau University of Science and Technology, Avenida Wai Long, Macau 999078, China

**Keywords:** ischemic stroke, natural compounds, traditional Chinese medicine, signaling pathways, targets

## Abstract

Ischemic stroke (IS) is a common neurological disorder associated with high disability rates and mortality rates. At present, recombinant tissue plasminogen activator (r-tPA) is the only US(FDA)-approved drug for IS. However, due to the narrow therapeutic window and risk of intracerebral hemorrhage, r-tPA is currently used in less than 5% of stroke patients. Natural compounds have been widely used in the treatment of IS in China and have a wide range of therapeutic effects on IS by regulating multiple targets and signaling pathways. The keywords “ischemia stroke, traditional Chinese Medicine, Chinese herbal medicine, natural compounds” were used to search the relevant literature in PubMed and other databases over the past five years. The results showed that JAK/STAT, NF-κB, MAPK, Notch, Nrf2, and PI3K/Akt are the key pathways, and SIRT1, MMP9, TLR4, HIF-α are the key targets for the natural compounds from traditional Chinese medicine in treating IS. This study aims to update and summarize the signaling pathways and targets of natural compounds in the treatment of IS, and provide a base of information for the future development of effective treatments for IS.

## 1. Introduction

Stroke is associated with the second leading cause of death and the third leading cause of disability among human diseases [1], and IS specifically accounts for over 80% of all stroke cases [2]. Due to the rapidly growing and aging population, IS incidence increases dramatically with age, As a result, this disease has a substantial impact on both afflicted families and society at large [3]. IS is characterized by localized ischemic and hypoxia necrosis of brain tissue caused by infarction and occlusion of cerebral arteries, which is often accompanied by significant physical and cognitive impairment [4]. IS can be treated by opening the occluded vessels as soon as possible to restore blood flow to the ischemic areas [5]. Recombinant tissue plasminogen activator (r-tPA) is the only US(FDA)-approved drug for IS treatment in the United States. The therapeutic time window for r-tPA is extremely limited, because it must be injected intravenously within 4.5 h of stroke onset. Furthermore, there is a substantial risk of hemorrhagic transformation, which may lead to additional difficulties [6]. In addition, thrombolysis with r-tPA is limited by slow reperfusion and is associated with significant bleeding risk, about 50% of patients who received this treatment develop cerebral ischemia/reperfusion injury (CIRI) [7], which can result in major consequences and long term disability for effective treatment of IS, new and more reliable therapeutic approaches are urgently needed.

IS is a complex pathological cascade reaction involving various pathological factors, including oxidative stress, inflammation, apoptosis, autophagy, and BBB damage. Oxidative stress, inflammation, and apoptosis are the critical factors in cerebral ischemic injury [8,9]. An imbalance in the amount of reactive oxygen species (ROS) is the cause of oxidative stress [10]. Under normal physiological conditions, the body maintains a dynamic ROS balance, during cerebral ischemia, a large accumulation of ROS leads to intracellular damage as well as mitochondrial damage, and cell transduction pathways are disrupted, inducing an apoptotic cascade reaction which in turn promotes the inflammatory response and the occurrence of apoptosis, further aggravating the oxidative damage of the organism [11], and finally leads to neuronal cell necrosis, senescence and apoptosis [9,12]. In the pathological process of IS, oxidative stress, inflammation, and apoptosis interact with each other and form a complex signaling network that plays a key role in the cerebral ischemic cascade [13]. The continuous exploration of intracellular signaling pathways leads to neuronal cell necrosis, senescence, and apoptosis.

Traditional Chinese medicine (TCM) has been practiced in China for thousands of years, and has gained wide clinical application [14], numerous clinical and laboratory investigations have been conducted over the last decades to confirm the effectiveness of TCM in the treatment of IS. According to the research, TCM demonstrated anti-IS activity and was shown to be safe and well-tolerated, TCM has various neuroprotective and repairing effects including maintaining blood–brain barrier (BBB) function, decreasing brain edema, regulating energy metabolism, promoting antioxidation, anti-inflammatory, and anti-apoptosis, reducing excitatory amino acid toxicity, enhancing neurogenesis, angiogenesis, and synaptogenesis [15]. The main advantage of TCM is that it often contains numerous components and affects many targets capable of producing additive or synergistic effects for treating IS. However, there is a lack of system review about the pathways and targets for TCM treating IS. This paper summarizes signaling pathways and potential therapeutic targets of natural compounds originated from TCM, to provide ideas for developing new anti-IS drugs.

## 2. The Signaling Pathways of Active Compounds in the Treatment of IS

### 2.1. JAK/STAT Signaling Pathway

The JAK/STAT signaling pathway is involved in various physiological processes, such as cell proliferation, differentiation, and apoptosis. The JAK protein tyrosine kinase family consists of JAKl, JAK2, JAK3, and Tyk2. To date, seven members of the STAT family have been ascertained: STAT1, STAT2, STAT3, STAT4, STAT5a, STAT5b, and STAT6 [16]. Extracellular signals such as cytokines and growth factors bind to corresponding receptors on the cell membrane, causing receptor dimerization and bringing receptor-coupled JAK kinases closer together, thus activating them through interactive tyrosine phosphorylation, which then phosphorylates STAT and transports it from the intracellular environment to the nucleus. STAT binds to the promoter region of the gene containing the γ-activation sequence, resulting in changes in the transcription and activity of DNA, which in turn affects essential cellular functions including cell growth, differentiation, and death [17]. After cerebral ischemia, ischemia and hypoxia can directly damage neurons and tissue cells in brain tissue, activate microglia and astrocytes in the ischemic area, and release inflammatory factors (IL-1, IL-6, TNF-α, ICAM-1α) and growth factors (EPO, ECF, PDGF), that can activate JAK/STAT signaling pathways [18]. It was shown that both JAK and STAT expression were upregulated in brain tissue after ischemia and then activated JAK-STAT phosphorylation, which significantly increased p-JAK and p-STAT protein expression and induced brain injury with brain edema, infarct size expansion, and neurological dysfunction. At the same time, downregulation of the JAK/STAT signaling pathway could reduce ischemic brain infarction, restore blood-brain barrier integrity and promote neurological recovery after cerebral ischemic injury [19,20].

**Figure 1:**
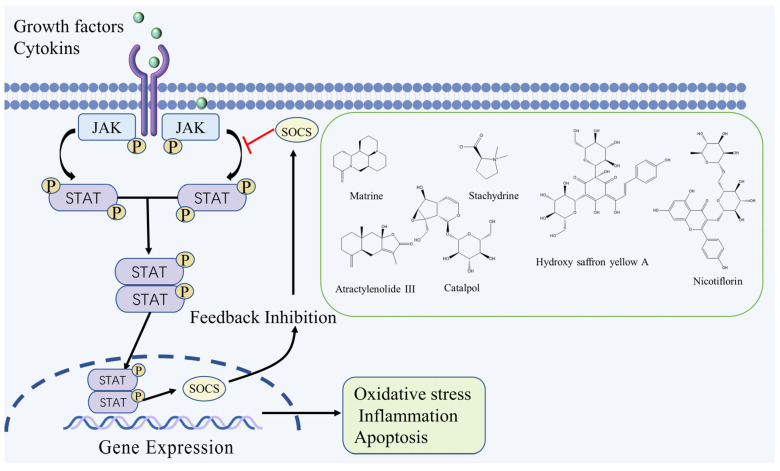
JAK/STAT signaling pathway and the chemical structure of natural compounds.

Matrine, an alkaloid that is extracted from *Sophora flavescens* Aiton., has been shown to reduce the expression of the p-JAK2 and p-STAT3 proteins and the number of apoptotic cells in the brain tissue of Middle Cerebral Artery Occlusion (MCAO) rats, and plays a neuroprotective role by inhibiting the activation of JAK-STAT signaling pathway and reducing the inflammatory response [21]. Hydroxy saffron yellow A is a flavonoid extracted from *Carthamus tinctorius* L., it can significantly down-regulate the expression of JAK2-mediated signaling due to ischemic injury, while significantly promoting the expression of SOCS3, which is a negative regulator of STAT3. By modulating the cross between JAK2/STAT3, Hydroxy saffron yellow A can confer neuroprotection against focal cerebral ischemia [22]. Catalpol, a terpenoid extracted from *Rehmannia glutinosa* (Gaertn.) Libosch. ex Fisch. & C. A. Mey., has multiple pharmacological activities, it can increase blood flow in ischemic brain tissues of MCAO rats, upregulate EPO and EPOR expression, promote STAT3 phosphorylation and inhibit VEGF mRNA expression, thus improving blood supply to ischemic brain tissues, reducing vascular permeability and promoting angiogenesis through the JAK2/STAT3 signaling pathway [23]. Nicotiflorin, a flavonoid extracted from *Carthamus tinctorius* L., can increase the protein expression level of Bcl-2 and downregulate the expression of p-JAK2, p-STAT3, caspase-3 and Bax, and inhibit the JAK2/STAT3 signaling pathway to alleviate apoptosis caused by cerebral ischemia-reperfusion injury (CIRI) [24]. Additionally, in vivo and in vitro experiments showed that Atractylenolide III and Stachydrine could exert antioxidant and anti-inflammatory effects by inhibiting the JAK2/STAT3 signaling pathway, and thus play a neuroprotective role [25,26]. The JAK/STAT signaling pathway and the chemical structure of natural compounds are shown in Figure 1.

### 2.2. NF-κB Signaling Pathway

The NF-κB signaling pathway is a classic signal transduction pathway mediated by cytokines. It plays an important role in several physiological and pathological activities, including inflammation, oxidative stress, endothelial cell injury, and cell death [27]. NF-κB is a significant transcriptional regulatory factor, comprising NF-κB1 (p50), NF-κB2 (p52), Rel A (p65), Rel B, and c-Rel [28]. Under normal conditions, NF-κB is inhibited and exists in the cytoplasm as a dimer in a complex with its inhibitory protein IκB. IκB can obscure the nuclear localization signal of NF-κB, making it inactivated. After the cerebral ischemic injury, cells are stimulated by factors such as inflammation and oxidation, and IκB proteins are degraded by phosphorylation, resulting in the dissociation of NF-κB dimers from the inactive complex to the activated state. Activated NF-κB migrates into the nucleus due to nuclear localization signal exposure and exerts its transcriptional regulatory role to induce the transcriptional synthesis and expression of relevant inflammatory factors, ultimately aggravating the degree of brain injury [29,30].

**Figure 2:**
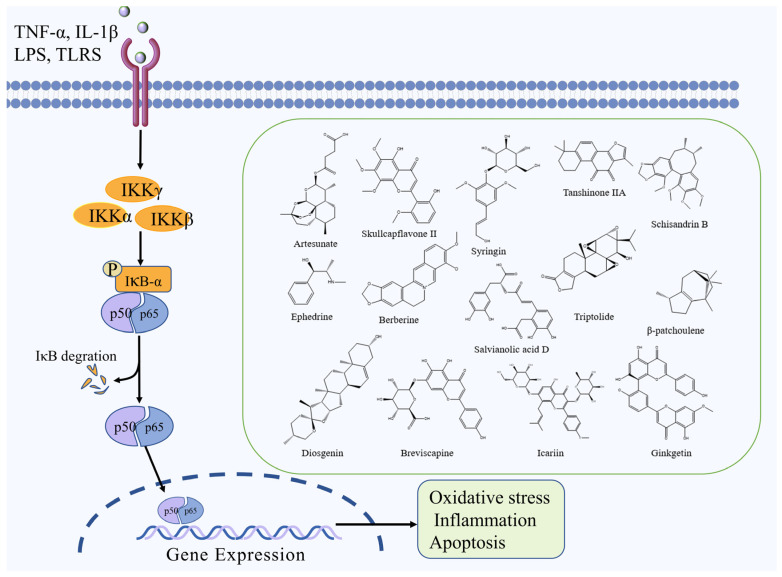
NF-κB signaling pathway and the chemical structure of natural compounds.

Artesunate, a derivative of artemisinin, reduces tissue damage caused by traumatic brain injury and protects MCAO mice from inflammatory injury by inhibiting NF-κB, releasing the pro-inflammatory cytokines IL-1β and TNF-α, reducing neutrophil infiltration, and inhibiting microglia activation [31]. Skullcapflavone II, a flavonoid from *Scutellaria baicalensis* Georgi, exerts protective effects against cerebral ischemia by inhibiting TLR4/NF-ĸB signaling pathway and suppressing mitochondrial apoptosis, inflammation, and oxidative stress [32]. Syringin, a lignan isolated from *Eleutherococcus senticosus* (Rupr. & Maxim.) Maxim., can promote FOXO3a phosphorylation and inhibit NF-κB nuclear translocation, which in turn reduces the levels of pro-inflammatory cytokines IL-1β, IL-6, TNF-α and MPO, and exerts a protective effect against ischemic brain injury by reducing the inflammatory response through the FOXO3a/NF-κB signaling pathway [33]. Schisandrin B, a lignan derivative isolated from *Schisandra chinensis* (Turcz.) Baill., can inhibit TLR4 expression and NF-κB activation and reduces TNF-α, IL-6 and IL-1β levels, exerts protective effects against cerebral ischemia by inhibiting TLR4/NF-κB signaling pathway [34]. Ephedrine, an alkaloid isolated from *Ephedra sinica* Stapf, has been shown to decrease oxidative stress, prevent inflammation, increase immunological function, and decrease CIRI, which may be due to their suppression of NF-κB-NLRP3 signaling [35]. Salvianolic acid D, a polyphenol component of *Salvia miltiorrhiza* Bunge, inhibits NF-κB activation and inflammatory factor release mediated by HMGB1-TLR4 signaling and attenuates HMGB1-mediated inflammatory response by inhibiting TLR4/MyD88/NF-κB signaling pathway [36]. Furthormore, other natural compounds such as triptolide, β-patchoulene, ginkgetin, tanshinone IIA, breviscapine, diosgenin, icariin, and berberine can also exert a protective effect against ischemic brain injury by inhibiting the NF-κB signaling pathway [37,38,39,40,41,42,43,44]. The NF-κB signaling pathway and the chemical structure of natural compounds are shown in Figure 2.

### 2.3. MAPK Signaling Pathway

Recent research demonstrated that the MAPK pathway plays an essential role in the initiation and progression of IS [45]. The MAPK family comprises conserved serine/threonine protein kinases in eukaryotes that function as crucial regulators of cell physiology and immune responses. MAPK transmits signals from the cytoplasm to the nucleus and activates various biological reactions, such as cell proliferation, differentiation, apoptosis, oxidative stress, inflammation, and innate immunity [46,47,48]. First, extracellular stimulation activates MAPK on the cell membrane via autophosphorylation. Once MAPK is activated, MAPK3 phosphorylates and activates MAPK2. MAPK2 then phosphorylates MAPK threonine/tyrosine residues, eventually activating and transferring MAPK into the nucleus, interacting with transcription factors such as c-Jun and c-Fos. Finally, MAPK upregulates the expression of target genes or acts on downstream kinases in the cytoplasm and regulates cellular activity [49,50]. Numerous experiments have shown that the MAPK signaling pathway is involved in multiple stages of cerebral ischemic and hypoxic injury. MAPK3 phosphorylation is inhibited during cerebral ischemic injury, and the application of MAPK pathway-specific inhibitors reduces phosphorylated MAPK3 expression and increases the number of cells in the ischemic area, suggesting that MAPK signaling pathway is involved in the protection of neurons after ischemia and plays an anti-modulatory role in cerebral ischemia-reperfusion [51].

**Figure 3:**
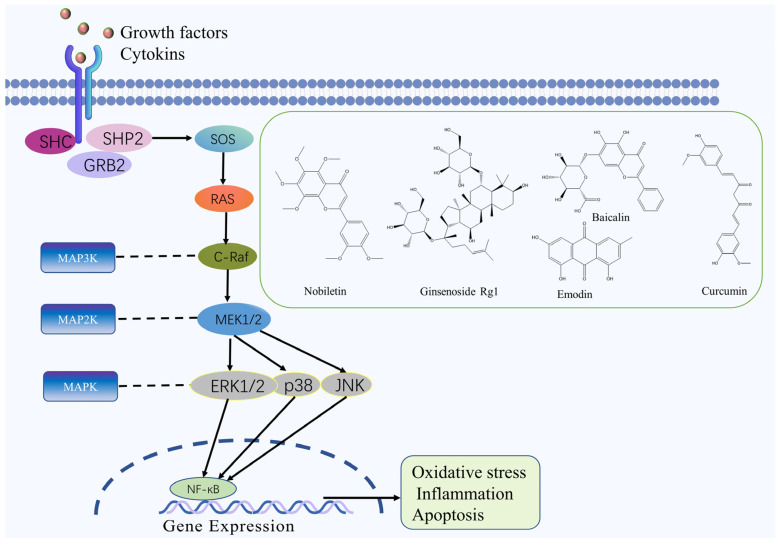
MAPK signaling pathway and the chemical structure of natural compounds.

Nobiletin, a flavonoid extracted from *Citrus reticulata* Blanco, can reduce ischaemic/reperfusion-induced brain apoptosis by upregulating Bcl-2 expression, downregulating Bax and caspase-3 expression, and reducing the levels of pro-inflammatory factors TNF-*α* and IL-6 and the expression of *p*-p38 and MAPAP-2 in MCAO rats. This mechanism is related to the MAPK signaling pathway [52]. Coriolus versicolor polysaccharides (CVP) can inhibit the phosphorylation of p38 MAPK, up-regulate Bcl-2 expression, down-regulate Bax and Caspase-3 activity, reduce the number of CIRI neuronal apoptosis; reduce the area of cerebral infarction, through the regulation of MAPK signaling pathway to achieve the role of protecting neuronal cells and restoring brain function [53]. Scrophularia ningpoensis polysaccharides can regulate the brain injury of CIRI rats by improving the antioxidant capacity of brain tissue, inhibiting the excessive production of inflammatory cytokines, inhibiting the expression of the JNK, p38, ERK, and other MAPK pathway proteins [54]. Emodin, a quinone isolated from *Rheum palmatum* L., can induce the expression of Bcl-2 and GLT-1 through the ERK-1/2 signaling pathway, inhibits neuronal apoptosis and ROS production, reduces glutamate toxicity, and alleviates nerve cell injury in a rat model of MCAO [55]. Furthermore, ginsenoside Rg1, baicalin and curcumin also have significant neuroprotective effects in IS by inhibiting the MAPK signaling pathway [56,57,58]. The MAPK signaling pathway and the chemical structure of natural compounds are shown in Figure 3.

### 2.4. Notch Signaling Pathway

Notch is a highly conservative signaling pathway that plays a critical role in cell proliferation, differentiation, and apoptosis [59], it is activated by ischemia and hypoxia in brain tissue during IS. The activated Notch pathway promotes the proliferation of neural stem cells and recovers the neural function defect after ischemia and promotes the neovascularization in the ischemic area, improves the ischemic and anoxic state of brain tissue, and effectively protects the recovery of neural function [60]. The Notch signaling pathway mostly comprises Notch receptors (Notch1~4), ligands (Jagged1/2 and Delta-like-1/3/4), and intracellular effector molecules (CSL) and Notch effector. Notch signaling is activated following Notch receptor-ligand binding on contacting cells [61]. The Notch receptor protein undergoes 3 cleavage and is released from the Notch intracellular domain (NICD) into the cytoplasm to form the NICD/CSL transcription activation complex, which enters the nucleus and binds to the transcription factor CSL, thereby activating the target genes of the transcriptional repressor family such as HES, HEY, HERP, etc. to play a biological role [62].

**Figure 4:**
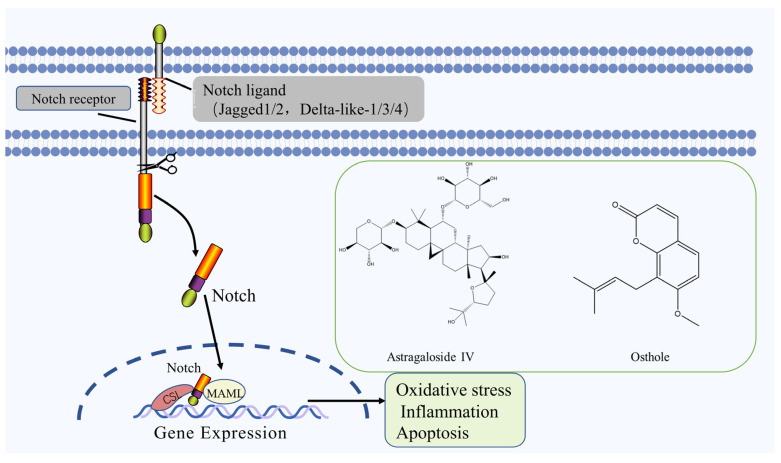
Notch signaling pathway and the chemical structure of natural compounds.

Astragaloside IV, the main saponin isolated from roots of *Astragalus penduliflorus subsp. mongholicus* (Bunge) X. Y. Zhu significantly reduced the infarct area in MCAO rats, and promoted cell proliferation and duct formation, which in turn promoted angiogenesis and had a protective effect against cerebral ischemic injury, which was closely related to the upregulation of miRNA-210 expression, induction of HIF-VEGF-Notch signaling pathway activation and inhibition of target gene ewitinA3 expression [63]. Osthole, a coumarin derivative isolated from fruits of *Cnidium monnieri* (L.) Cusson., can significantly reduce the volume of cerebral infarction, reduce apoptosis, increase the expression of target proteins Notch1, Hes-5 and NICD by acting on the Notch pathway, and play a protective role for neurons [64]. The Notch signaling pathway and the chemical structure of natural compounds are shown in Figure 4.

### 2.5. Nrf2 Signaling Pathway

The Nrf2 signaling pathway plays a significant role in the occurrence and development of IS, and it can regulate the ability of cells to resist oxidative stress and protect brain tissue [65]. Nrf2 belongs to the CNC basic leucine zipper transcriptional activator family, containing seven highly conserved functional structures. When stimulated by oxygen radicals, each of these structural domains plays a role in regulating the activation of Nrf2 and initiating the transcription of downstream genes, thereby protecting the cell from damage. In the resting state, Nrf2 can be coupled with its inhibitory factors, so that the antioxidant capacity of the cell is at the most basic level. After ROS attack, Nrf2 is decoupled and released into the cytoplasm in large quantities. Moreover, Nrf2 can bind to ARE and initiate the transcription of downstream endogenous protective genes and phase II detoxifying enzymes, such as HO-1 and NQO1, and regulates antioxidant enzymes including SOD, CAT, GSH-Px, and GST, which are key in cell self-protection [66,67,68,69,70].

**Figure 5:**
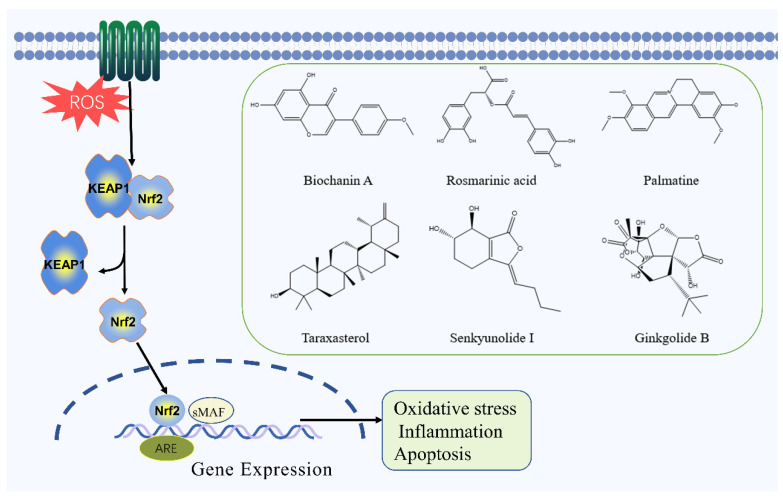
Nrf2 signaling pathway and the chemical structure of natural compounds.

Biochanin A, the main flavonoid component of *Trifolium pratense* L., promotes the nuclear translocation of Nrf2 and induces the expression of HO-1 by regulating the Nrf2/HO-1 signaling pathway, it protects the rat brain from ischemic injury through antioxidant and anti-inflammatory effects [71]. Rosmarinic acid, a water-soluble polyphenol compound widely found in the plant species of Lamiaceae and Boraginaceae [72], can up-regulate Bcl-2 and down-regulate the level of Bax and Caspase-3 to exert its anti-apoptotic effect. This effect is related to activating the Nrf2/HO-1 pathway and inhibiting the p53 gene [73]. Adenosine monophosphate (AMPK) is an important intracellular metabolic and stress receptor, and is a key regulatory protein of autophagy. Palmatine, the main alkaloid of *Coptis chinensis* Franch., can reduce oxidative stress, inflammatory response, and neuronal apoptosis in MCAO mice by activating the AMPK/ Nrf2 pathway [74]. Taraxasterol, the main terpenoid ingredient of *Taraxacum mongolicum* Hand.-Mazz., can significantly inhibit the generation of ROS and MDA in hippocampal neurons induced by OGD/R, leading to a decrease in caspase-3 and Bcl-2 expression, and a concurrent increase in the expression of Bax, HO-1, NQO-1, and GPX-3. Taraxasterol can protect hippocampal neurons from OGD/R-induced injury by activating the Nrf2 signaling pathway [75]. In addition, senkyunolide I and ginkgolide B can also protect brain tissue from ischemic injury by inhibiting the Nrf2 signaling pathway [76,77]. The Nrf2 signaling pathway and the chemical structure of natural compounds are shown in Figure 5.

### 2.6. PI3K/Akt Signaling Pathway

There are many experimental studies on the regulatory role of the PI3K/Akt signaling pathway in IS [78,79,80]. The PI3K/Akt/mTOR signaling pathway plays a neuroprotective role in ischemic reperfusion injury by upregulating the expression of PI3K, p-Akt, and p-mTOR in brain tissue, which significantly reduces the brain infarct size in MCAO rats and the pathological changes of brain tissue, thus alleviating CIRI [81]. PI3K can be further divided into PI3KⅠ, PI3KⅡ, and PI3KⅢ according to its structure and substrate specificity [82]. Akt is an essential active signaling target downstream of PI3K and is a serine/threonine protein kinase [83,84]. PI3K activation leads to the formation of PIP3 on the plasma membrane, which induces a conformational change in Akt. As a result, Akt transfers to the cell membrane and exposes its two major phosphorylation sites, Thr308 and Ser473. PDK1 phosphorylates Thr308 and PDK2 phosphorylates Ser473, resulting in the full activation of Akt that then can regulate cell proliferation, differentiation, and apoptosis by activating or inhibiting downstream signaling factors [85]. The activation of the PI3K/Akt signaling pathway participates in the pathological process of cerebral ischemia, promotes the proliferation and differentiation of neural stem cells, and protects neural cells from ischemia-related injury and death [86].

**Figure 6:**
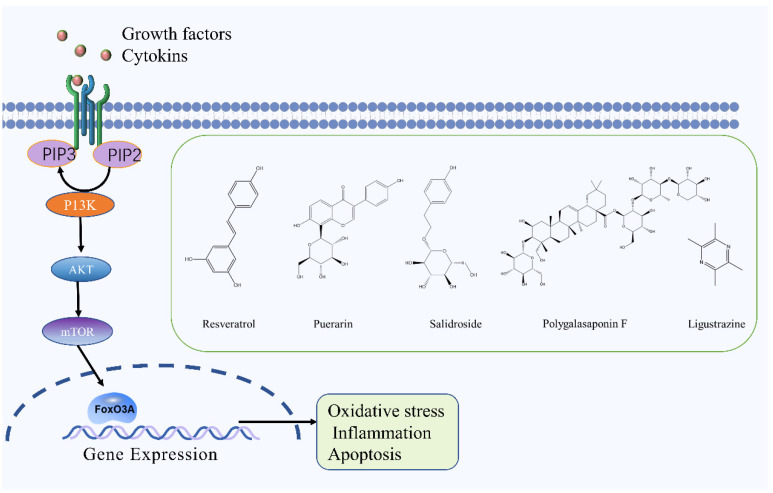
PI3K/Akt signaling pathway and the chemical structure of natural compounds.

Resveratrol is a natural polyphenol isolated from plants such as *Reynoutria japonica* Houtt. and *Vitis vinifera* L., it reduces the expression of IL-1β, COX-2 and TNF-α by stimulating the PI3K/Akt signaling pathway as well as decreasing infiltration of neutrophils, thereby reducing the inflammatory response in rats with ischemic stroke [87]. Ligustrazine, the main alkaloid ingredient of *Ligusticum chuanxiong* Hort., can significantly increase the levels of p-Akt and p-eNOS in the brain tissue of MCAO rats, and play a neuroprotective role on the brain of ischaemic/reperfusion injury rats by stimulating the PI3K/Akt pathway [88]. Polygalasaponin F, the main terpenoid of *Polygala tenuifolia* Willd., can downregulate the expression of Bcl-2/Bax and caspase-3 in PC12 cells and prevent OGD/R-induced injury by stimulating the PI3K/Akt signaling pathway [89]. Puerarin, a flavonoid isolated from *Puerariae Lobata* (Willd.) Ohwi, can significantly increase the expression of Akt1, GSK-3β, and MCL-1 p62 as well as decrease caspase-3 expression levels in MCAO rats. These findings indicate that puerarin can regulate the neuroprotective mechanism of autophagy via the PI3K/Akt1/GSK-3β/MCL-1 signaling pathway [90]. In addition, Panax notoginseng saponins and salidroside can also prevent ischemic injury by stimulating the PI3K/Akt signaling pathway [91,92]. The PI3K/Akt signaling pathway and the chemical structure of natural compounds are shown in Figure 6.

## 3. The Target Protein of Natural Compounds in the Treatment of IS

### 3.1. SIRT1

SIRT1 is a nicotinamide adenine dinucleotide-dependent histone deacetylase with deacetylation of various histones and non-histones [93], which can regulate pathological processes such as oxidative stress, inflammatory response, and apoptosis by regulating FOXO, NF-κB PARP-1, PGC-1, PPAR-γ, and eNOS deacetylation, exerting a role in regulating pathological processes such as oxidative stress, inflammatory response, and apoptosis [94]. In SIRT1-deficient mice, CIRI is manifested by increased levels of inflammation, oxidative stress, and apoptosis, suggesting that SIRT1 may play a neuroprotective role [95]. Ginsenosides activate SIRT1 protein expression in the ischemic penumbra of MCAO rats, and SIRT1 can directly deacetylate the p65 subunit of NF-κB and reduce its acetylation level, thereby inhibiting the transcriptional activity of NF-κB and the expression of IL-1β, IL-6, and TNF-α, and reduce the ischemic injury and neurological deficits in MCAO rats [96]. Magnolol (a phenolic compound derived from *Magnolia officinalis* Rehd. Et Wils) and Salvianolic acid B (a phenolic compound derived from *Salvia miltiorrhiza* Bunge.) can regulate brain injury induced by cerebral ischemia by activating SIRT1, deacetylating to inhibit Ac-FOXO1 expression, and suppressing inflammatory cytokines and apoptosis [97,98]. Calycosin-7-*O*-*β*-*D*-glucoside, a flavonoid isolated from *Astragalus penduliflorus subsp. mongholicus var. dahuricus* (Fisch. ex DC.) X. Y. Zhu, can attenuate OGD/R-induced oxidative stress and neuronal apoptosis by activating SIRT1 and upregulating FOXO1 and PGC-1 α expression [99]. Moreover, the inhibitor of Sirt1 can reverse these neuroprotective effects.

### 3.2. MMP9

MMP9 is a member of the zinc-dependent protein hydrolase family and can degrade extracellular matrix, including collagen IV, laminin, and fibronectin [100]. MMP9 expression increased during cerebral ischemia [101]. Up-regulated MMP9 destroys the structural integrity of brain microvessels and the blood-brain barrier by degrading the extracellular matrix, resulting in secondary brain edema and brain injury [102], while knockout of MMP9 in mice or the use of MMP9 inhibitors can reduce brain edema [103]. Therefore, MMP9 is expected to be a target for treating ischemic brain injury. TIMP1 is an endogenous inhibitor that regulates the activity of MMP9 and can inhibit the activity of MMP9 through non-covalent binding to the catalytic domain of MMP9. The imbalance between MMP-9 and TIMP-1 can lead to secondary brain damage. Icariside II (a flavonoid derived from *Epimedium brevicornu* Maxim.) and ursolic acid (a pentacyclic triterpene derived from many plants, such as *Scleromitrion diffusum* (Willd.) R. J. Wang and *Actinidia chinensis* Planch.) could further inhibit neuronal apoptosis by regulating the balance of MMP9/TIMP1, thereby significantly improving the ischemia-reperfusion induced BBB disruption in MCAO rats, preventing cerebral ischemia-reperfusion injury [104,105]. Calycosin-7-O-β-D-glucoside (a flavonoid extracted from *Astragalus penduliflorus subsp. mongholicus var. dahuricus* (Fisch. ex DC.) X. Y. Zhu) and oxymatrine (an alkaloid derived from *Sophora flavescens* Aiton) can reduce the expression of MMP9 protein by downregulating the expression of CAV1, thereby improving the integrity of the BBB after CIRI [106,107].

### 3.3. TLR4

TLR4, also known as CD284, is a transmembrane protein in the Toll-like receptor family [108]. During cerebral ischemia, damaged tissues and cells release damage-associated molecular patterns (DAMPs), such as S100 protein and HMGB1, DAMPs can bind and activate TLR4, TLR4 can activate NF-κB through MyD88 and TRIF pathways, thereby activating inflammatory responses and aggravating brain tissue damage [109,110]. Compared with wild-type mice, the infarct area and volume of TLR4 knockout mice after ischemia/reperfusion are obviously smaller, and the neurological deficit is improved, indicating TLR4 may be one of the targets for the treatment of cerebral ischemia injury [111]. Gentianine, an alkaloid isolated from *Gentiana scabra* Bunge., can inhibit and attenuate the expression of TLR4, MyD88 mRNA, and nuclear translocation of NF-κB in brain tissue, as well as the levels of IL-1β, TNF-α, and IL-6 in serum, suggesting that gentianine may reduce brain tissue injury due to ischemia/reperfusion by inhibiting TLR4 pathway-mediated inflammatory response [112]. Procyanidins, polyphenols extracted from grape seeds, suppress the activation of the NLRP3 inflammasome by inhibiting the expression of TLR4, thereby reducing the inflammatory response and improving cerebral ischemia-reperfusion injury [113].

### 3.4. HIF-α

HIF-1α is a transcription factor that is widely distributed in mammals under hypoxic conditions and can activate a variety of hypoxia-response genes (HRGs) expression to regulate the oxygen homeostasis and energy metabolism balance of cells and organism [114]. HIF-1α-induced gene expression can improve glucose transport and blood circulation in the ischemic penumbra after cerebral infarction, mediating hypoxia tolerance after hypoxia, regulating the immune response, and has a significant protective effect on ischemia-hypoxic neurons [115]. In addition, HIF-1α can inhibit PTP by reducing ROS and Ca^2+^ generated during cerebral ischemia-reperfusion, thereby reducing brain cell apoptosis [116], and can also activate various brain protective signaling pathways, such as PI3K/AKT and JAK2/STAT3 pathway to improve mitochondrial respiratory function to protect brain tissue after ischemia-reperfusion [117]. Catalpol (an iridoid glycoside extracted from *Rehmannia glutinosa* (Gaertn.) Libosch. ex Fisch. & C. A. Mey.) and Cardamonin (a chalcone component extracted from the seeds of *Amomum villosum* Lour..) activates the HIF-1α/VEGF signaling pathway in rats with ischemia-reperfusion injury, and upregulate the protein expression of HIF-1α and VEGF, thereby increasing cerebral microvascular density and promoting intracerebral revascularization, and promoting angiogenesis, neural repair and functional recovery in MCAO rats [118,119].

## 4. Conclusions and Future Aspects

IS is a serious life-threatening disease associated with high rates of disability and mortality. Due to the rapid onset of the disease, there are many delayed factors in the treatment process, making early thrombolysis challenging to implement, thus affecting the outcome of neural health and functioning post-stroke. In recent years, a large amount of literature has shown that TCM can significantly improve IS with few side effects, demonstrating TCM’s potential benefits and significant potential for future development. TCM can regulate the signaling pathways to treat IS, such as JAK/STAT, NF-κB, MAPK, Notch, Nrf2, PI3K/Akt, which maintain BBB function, decrease brain edema, regulate energy metabolism, promote antioxidation, anti-inflammatory, and anti-apoptosis, reducing excitatory amino acid toxicity, enhancing neurogenesis, angiogenesis, and synaptogenesis.

By summarizing and analyzing the natural compounds of traditional Chinese medicine that treat IS, we found that there are mainly flavonoids, alkaloids, polysaccharides, saponins, polyphenols, and terpenoids. The mechanism is mainly related to anti-oxidation, anti-inflammation, anti-apoptosis, and improving the permeability of BBB. The regulation effect of natural compounds from TCM on IS-related signaling pathways is shown in Table 1. Flavonoids are a type of secondary metabolite produced in many plants and have beneficial biological properties such as strong antioxidants and anti-inflammatory [120]. Flavonoids often have higher free oxygen radical scavenging activity. The more hydroxyl substituents in the parent nucleus of natural flavonoids, the stronger their free oxygen radical scavenging activity, especially the ortho hydroxyl substitution can greatly improve their activity. the catechol structure on the benzene ring is an important active group [121]. Alkaloids are an ubiquitous class of nitrogenous organic compounds in nature, most of which have complex ring structures, and contained nitrogen elements. Studies show that alkaloids are active ingredients in many Chinese herbal medicines and have biological properties such as anti-tumor, anti-inflammatory, anti-bacterial, antiviral, and insecticidal properties [122]. The nitrogen-containing heterocycles are the key active group for alkaloids in the treatment of IS. Polysaccharides are composed of more than 10 monosaccharide molecules connected by α- or β-glycoside bonds [123]. Polysaccharides have many pharmacological properties such as immunomodulatory, antioxidant, antiviral, anti-tumor, and anti-diabetic properties, and they are significant to the prevention and treatment of various diseases [124,125,126,127,128,129]. Differences in the branching degree of polysaccharides, the type of glycosidic bonds and the composition of glycosyl groups, and the substituent groups can affect the activity of polysaccharides. As one of the basic components of polysaccharides, the content of uronic acid is directly related to the ability to scavenge free radicals and antioxidant activity [130]. Saponins are a class of glycosides found commonly in plants. Recent studies have shown that saponins have anti-tumor, anti-inflammatory, immunomodulatory, antiviral, and anti-fungal properties. According to the chemical structure of saponins, they can be divided into triterpenoid saponins and steroidal saponins [131,132]. The structure of glycosides, the monosaccharide composition, and the structure of sugar chains have important effects on the activity of saponins. Polyphenols are secondary metabolites of plants with strong antioxidant capacity [133]. The carboxyl and carbonyl groups in their structure directly determine their antioxidant activity. Terpenes are the largest secondary metabolites of plants. Terpenoids can be divided into monoterpenes, sesquiterpenes, diterpenes, sesquiterpenes, triterpenes, tetraterpenes, and polyterpenes according to the number of isoprene units in the molecule [134]. Terpenoids have anti-inflammatory, antibacterial, antioxidant and antitumor effects [135]. Iridoid terpenes are one of monoterpenes. The anti-inflammatory group is the p-methoxy cinnamaldehyde group [136]. It is found that the physiological activity of naturalcompounds is closely related to their chemical structure, and the distribution of the mother nucleus and substituent may affect their pharmacological activity. The relationship between the structure and activity of natural compounds for the treatment of cerebral ischemia is analyzed and summarized, which will play a good role in promoting the activity prediction and structure optimization of natural compounds, and to provide a research basis for the development of new drugs with higher activity for the treatment of cerebral ischemia in the future.

Despite the positive therapeutic effect of TCM on IS, there are still numerous challenges to overcome. Most of the research on TCM in IS models is still in the primary experimental stage, with few clinically applicable products. Furthermore, only known signaling pathways and related protein targets have been shown to play an important role in IS. However, there may be other pathway targets that have not yet been discovered. A comprehensive strategy combining metabolomics and network pharmacology is an effective tool to discover the targets and signaling pathways of TCM against cerebral ischemia-reperfusion. This strategy has been applied to TCM research, including drug target discovery, efficacy evaluation and mechanism research [137,138,139]. Futhermore, the efficacy and safety of TCM intervention on IS need to be verified through rigorous and high-quality clinical trials.

## Figures and Tables

**Table 1:** Regulation effect of natural compounds from TCM on IS-related signaling pathways.

Natural Compounds	Categories	Plants	Experiments Model		Mechanisms	Signaling Pathways	Ref.
			In Vivo	In Vitro			
*Matrine*	Alkaloid	*Sophora flavescens* Aiton.	MCAO rats	--	↑: SOD, ↓: MDA, p-JAK2, p-STAT3	JAK2/STAT3	[21]
Hydroxy saffron yellow A	Flavonoid	*Carthamus tinctorius* L.	MCAO rats	--	↑: SOCS3 ↓: p-JAK2, p-STAT3	JAK2/STAT3	[22]
Catalpol	Terpenoid	*Rehmannia glutinosa* (Gaertn.) Libosch. ex Fisch. & C. A. Mey.	MCAO rats	--	↑: VEGF, EPO, EPOR ↓: p-JAK2, p-STAT3	JAK2/STAT3	[23]
Nicotiflorin	Flavonoid	*Carthamus tinctorius* L.	MCAO rats	--	↑: Bcl-2 ↓: p-JAK2, p-STAT3, caspase-3, Bax	JAK2/STAT3	[24]
Atractylenolide III	Terpenoid	*Atractylodes macrocephala* Koidz.	MCAO rats	OGD/R cells	↓: IL-1β, TNF-α, IL-6, Drp1, p-JAK2, p-STAT3	JAK2/STAT3	[25]
Stachydrine	Alkaloid	*Leonurus japonicus* Houtt.	MCAO rats	OGD/R cells	↑: SOD ↓: p-65, p-iκB, p-JAK2, p-STAT3, MDA, IL-1β, TNF-α	JAK2/STAT3	[26]
Artesunate	Terpenoid	*Artemisia annua* L.	MCAO mice	--	↑: IκB ↓: IL-1β, TNF-α, NF-κB	NF-κB	[31]
Skullcapflavone II	Flavonoid	*Scutellaria baicalensis* Georgi	MCAO rats	--	↑: SOD, GSH, VEGF, Ang-1,Tie-2, ↓: MDA, IL-1β, TNF-α, IL-6, caspase-3 and -9, NF-ĸb, TLR4	NF-κB	[32]
Syringin	Saponin	*Eleutherococcus senticosus* (Rupr. & Maxim.) Maxim.	MCAO rats	--	↑: p-FOXO3a ↓: NF-κB, IL-1β, IL-6, TNF-α, MPO	NF-κB	[33]
Schisandrin B	Lignan	*Schisandra chinensis* (Turcz.) Baill.	MCAO rats	--	↓: NF-κB, TLR4, IL-1β, IL-6, TNF-α	NF-κB	[34]
Ephedrine	Alkaloid	*Ephedra sinica* Stapf *Ephedra sinica* Stapf	MCAO rats	--	↑: Bcl-2 ↓: IL-1β, TNF-α, IL-6, Bax, NO, p-NF-κB	NF-κB	[35]
Berberine	Alkaloid	*Coptis chinensis* Franch.	MCAO rats	--	↑: SOD, GSH-Px, CD4+, CD8 ↓: NO, TNF-α, IFN-β, IL-6, NF-κB p65, NLRP3, ASC, caspase-3	NF-κB	[44]
Salvianolic acid D	Polyphenol	*Salvia miltiorrhiza* Bunge	MCAO rats	OGD/R cells	↑: Bcl-2 ↓: Bax, Cyt c, caspase-3 and -9, TLR4, MyD88, TRAF6, NF-κB, HMGB1	NF-κB	[36]
Triptolide	Terpenoid	*Tripterygium wilfordii* Hook. f.	MCAO rats	--	↓: NF-κBp65, PUMA, caspase-3	NF-κB	[37]
β-patchoulene	Terpenoid	*Pogostemon cablin* (Blanco) Benth.	MCAO rats	--	↑: IκBα,SOD, GSH-Px, Bcl-2 ↓: NF-κBp65, TLR4, caspase-3, Bax, TNF-α, IFN-β, IL-6	NF-κB	[38]
Ginkgetin	Flavonoid	*Ginkgo biloba L.*	MCAO rats	--	↑: Bcl-2 ↓: LC3-II/LC3-I, DRAM, Beclin 1, cathepsin B, cathepsin D, DRAM, PUMA, Beclin 1, p53, Bax	NF-κB	[39]
Tanshinone IIA	Terpenoid	*Salvia miltiorrhiza* Bunge	MCAO rats	OGD/R cells	↑: SOD ↓: MDA, TNF-α, IL-1β, IL-6, p-iκB, p-p65	NF-κB	[40]
Breviscapine	Flavonoid	*Erigeronbreviscapus* (Vant.) Hand.-Mazz.	MCAO rats	--	↑: SOD, GSH-Px ↓: MDA, IL-6, IL-1β, TNF-α, PARP-1, COX2, iNOS, p65	NF-κB	[41]
Diosgenin	*Saponin*	*Dioscorea zingiberensis* C. H. Wright	MCAO rats	OGD/R cells	↑: HIKESHI, HSP70, IκBα ↓: TNF-α, IL-1β, IL-6, NF-κB	NF-κB	[42]
Icariin	Flavonoid	*Epimedium brevicornum* Maxim.	MCAO rats	--	↑: PPARα,PPARγ, IκBα ↓: TNF-α, IL-1β, IL-6, NF-κB	NF-κB	[43]
Berberine	Alkaloid	*Coptis chinensis* Franch.	MCAO rats	--	↑: SOD, GSH-Px, CD4+, CD8 ↓: NO, TNF-α, IFN-β, IL-6, NF-κB p65, NLRP3, ASC, caspase-3	NF-κB	[44]
Nobiletin	Flavonoid	*Citrus reticulata* Blanco	MCAO rats	--	↑: Bcl-2, IL-10, ↓: TNF-α, IL-6, caspase-3, Bax, p-p38, MAPKAP-2	MAPK	[52]
Coriolus versicolor polysaccharides	Polysaccharide	*Coriolus versicolor* (L. ex Fr.) Quel	MCAO rats	--	↑: Bcl-2, IL-10, ↓: Bax, TNF-α, IL-1β, caspase-3, p38 MAPK	MAPK	[53]
Scrophularia ningpoensis polysaccharides	Polysaccharide	*Scrophularia ningpoensis* Hemsl.	MCAO rats	--	↑: p-ERK, SOD ↓: p-JNK, p-p38, TNF-α, IL-1β, MDA, NO, NOS	MAPK	[54]
Emodin	*Quinone*	*Rheum palmatum* L.	--	OGD/R cells	↑: p-ERK-1/2, GLT-1, Bcl-2 ↓: caspase-3	MAPK	[55]
Ginsenoside Rg1	Terpenoid	*Panax ginseng* C. A. Mey.	MCAO rats	--	↑: Bcl-2 ↓: p-JNK, p-p38, caspase-3, Bax	MAPK	[56]
Baicalin	Flavonoid	*Scutellaria baicalensis* Georgi	--	OGD/R cells	↑: MAPK, ERK, MAP2, Bcl ↓: Bax, caspase-3 and -9	MAPK	[57]
Curcumin	Polyphenol	*Curcuma longa* L.	MCAO rats	--	↓: LC3-II/LC3-I, IL-1, TLR4, p-38, p-p38	MAPK	[58]
Astragaloside IV	*Saponin*	*Astragalus penduliflorus subsp. mongholicus var. dahuricus* (Fisch. ex DC.) X. Y. Zhu	MCAO rats	--	↑: HIF-1α, VEGF, Notch, DLL4	Notch	[63]
Osthole	Coumarin	*Cnidium monnieri* (L.) Cusson	MCAO rats	--	↑: Bcl-2, Notch, NICD, Hes 1 ↓: Bax, caspase-3,	Notch	[64]
Biochanin A	Flavonoid	*Trifolium pratense L*.	MCAO rats	--	↑: SOD, GSH-Px, HO-1, Nrf2 ↓: MDA	Nrf2	[71]
Rosmarinic acid	Polyphenol	*Rosmarinus officinalis* L.	MCAO rats	--	↑: Bcl-2, HO-1, Nrf2, SOD ↓: MDA, Bax	Nrf2	[73]
Palmatine	Alkaloid	*Coptis chinensis* Franch	MCAO rats	OGD/R cells	↑: Bcl-2, HO-1, Nrf2, SOD, CAT, p-AMPK ↓: MDA, Bax, TNF-α, IL-1β, IL-6	Nrf2	[74]
Taraxasterol	Terpenoid	*Taraxacum mongolicum* Hand.-Mazz.		OGD/R cells	↑: HO-1, NQO-1, GPx-3, Nrf2, Bcl-2 ↓: ROS, MDA, Bax	Nrf2	[75]
Senkyunolide I	Terpenoid	*Ligusticum chuanxiong* Hort.	MCAO rats	--	↑: SOD, Erk1/2, Nrf2, NQO1, Bcl-2 ↓: MDA, caspase-3, caspase-9, Bax	Nrf2	[76]
Ginkgolide B	Terpenoid	*Ginkgo biloba* L.	MCAO rats	OGD/R cells	↑: SOD, p-Akt, HO-1, Nqo1p-Nrf2 ↓: ROS	Nrf2	[77]
Resveratrol	Polyphenol	*Reynoutria japonica* Houtt.	MCAO rats	--	↑: p-AKT ↓: IL-1β, TNFα, COX2, MPO	PI3K/Akt	[87]
Ligustrazine	Alkaloid	*Ligusticum chuanxiong* Hort.	MCAO rats	OGD/R cells	↑: p-eNOS, p-AKT	PI3K/Akt	[88]
Polygalasaponin F	Terpenoid	*Polygala tenuifolia* Willd.		OGD/R cells	↑: p-AKT, Nrf2, HO-1 ↓: Bcl-2/Bax caspase-3	PI3K/Akt	[89]
Puerarin	Flavonoid	*Puerariae Lobata* (Willd.) Ohwi	4-vessel occlusion rats	--	↑: p-GSK-3β, MCL-1, p-AKT ↓: caspase-3	PI3K/Akt	[90]
Panax notoginseng Saponins	Saponin	*Panax notoginseng* (Burkill) F. H. Chen ex C. H. Chow	--	OGD/R cells	↑: p-AKT, Nrf2, HO-1 ↓: ROS	PI3K/Akt	[91]
Salidroside	Polyphenol	*Rhodiola rosea* L.	MCAO rats	--	↑: p-Akt ↓: IL-6, IL-1β, TNF-α, CD14, CD44, iNOs	PI3K/Akt	[92]

↑: up regulation; ↓: down regulation.

## Abbreviations

The following abbreviations are used in this manuscript:

IS	Ischemic stroke
r-tPA	Recombinant tissue plasminogen activator
FDA	Food and drug administration
US	United States
BBB	Blood–brain barrier
TCM	Traditional chinese medicine
CIRI	Cerebral ischemia/reperfusion injury
ROS	Reactive oxygen species
JAK	Janus Kinase
STAT	Signal Transducer And Activator Of Transcription
IL-1β	Interleukin-1β
TNF-α	Tumor necrosis factor-α
IL-6	Interleukin-6
ICAM-1α	Intercellular cell adhesion molecule-1 α
EPO	Erythropoietin
ECF	Epidermal Growth Factor
PDGF	Platelet-derivedgrowthfactorreceptor
MCAO	Middle Cerebral Artery Occlusion
NF-κB	Nuclear Factor Kappa-B
TLR4	Toll Like Receptor 4
FOXO3a	Forkhead box O3
MPO	Myeloperoxidase
NLRP3	NOD-like receptor protein 3
HMGB1	High Mobility Group Box 1
MyD88	Myeloiddifferentiationfactor88
MAPK	Mitogen-Activated Protein Kinase
CytC	Cytochrome c
Bcl-2	B-cell lymphoma-2
Bax	Bcl2-associated x protein
JNK	Jun N-Terminal Kinase
ERK	Extracellular Signal-Regulated Kinase
CSL	CBF1/suppressor of hairless/Lag
NICD	Notch intracellular domain
HIF	Hypoxia-inducible factor
VEGF	Vascular endothelial growth factor
Nrf2	Nuclear Factor E2-Related Factor 2
NQO1	Nad(p)h quinone oxidoreductase
HO-1	Heme oxygenase-1
SOD	Superoxide dismutase
CAT	Catalase
GPX-Px	Glutathione peroxidase
GSH	Reduced glutathione
AMPK	Adenosine monophosphate
OGD/R	Oxygen-Glucose Deprivation/Re-Oxygenation
MDA	Malondialdehyde
PI3K	Phosphoinositide 3-kinase
Akt	Protein kinase B
mTOR	mammalian target of rapamycin
SIRT1	Silent mating type information regulation 2 homolog-1.
MMP9	Matrix Metalloproteinase 9
